# Carvacrol, a Food-Additive, Provides Neuroprotection on Focal Cerebral Ischemia/Reperfusion Injury in Mice

**DOI:** 10.1371/journal.pone.0033584

**Published:** 2012-03-16

**Authors:** Hailong Yu, Zeng-Li Zhang, Jing Chen, Aijie Pei, Fang Hua, Xuanchen Qian, Jinjiang He, Chun-Feng Liu, Xingshun Xu

**Affiliations:** 1 Department of Neurology, The Second Affiliated Hospital of Soochow University, Suzhou City, Jiangsu Province, People's Republic of China; 2 Institute of Neuroscience, Soochow University, Suzhou City, Jiangsu Province, People's Republic of China; 3 Department of Toxicology, School of Public Health, Soochow University, Suzhou City, Jiangsu Province, People's Republic of China; 4 Department of Emergency Medicine, Brain Research Laboratory, Emory University School of Medicine, Atlanta, Georgia, United States of America; Julius-Maximilians-Universität Würzburg, Germany

## Abstract

Carvacrol (CAR), a naturally occurring monoterpenic phenol and food additive, has been shown to have antimicrobials, antitumor, and antidepressant-like activities. A previous study demonstrated that CAR has the ability to protect liver against ischemia/reperfusion injury in rats. In this study, we investigated the protective effects of CAR on cerebral ischemia/reperfusion injury in a middle cerebral artery occlusion mouse model. We found that CAR (50 mg/kg) significantly reduced infarct volume and improved neurological deficits after 75 min of ischemia and 24 h of reperfusion. This neuroprotection was in a dose-dependent manner. Post-treatment with CAR still provided protection on infarct volume when it was administered intraperitoneally at 2 h after reperfusion; however, intracerebroventricular post-treatment reduced infarct volume even when the mice were treated with CAR at 6 h after reperfusion. These findings indicated that CAR has an extended therapeutic window, but delivery strategies may affect the protective effects of CAR. Further, we found that CAR significantly decreased the level of cleaved caspase-3, a marker of apoptosis, suggesting the anti-apoptotic activity of CAR. Finally, our data indicated that CAR treatment increased the level of phosphorylated Akt and the neuroprotection of CAR was reversed by a PI3K inhibitor LY-294002, demonstrating the involvement of the PI3K/Akt pathway in the anti-apoptotic mechanisms of CAR. Due to its safety and wide use in the food industry, CAR is a promising agent to be translated into clinical trials.

## Introduction

Stroke is one of the three leading causes of death and disability and ischemic stroke accounts for the majority of stroke [1]. Ischemic stroke results from the occlusion of brain blood vessels, while the middle cerebral artery is mostly involved in ischemic stroke. Middle cerebral artery occlusion (MCAO) usually causes substantial decreases in local cerebral blood flow, thus reducing perfusion pressure and blood oxygen content, and resulting in an interdependent series of neurotoxic events, including glutamate excitotoxicity, Ca^2+^ overload, oxidative stress, nitric oxide (NO) production and inflammation [2], [3]. These events after cerebral ischemia injury finally cause neuronal cell death like apoptosis, necrosis, necroptosis and autophagy [4], [5]. In the past several decades, pathophysiological process of cerebral ischemia injury was well advanced, however, only intravenous recombinant tissue plasminogen activator (tPA) therapy has been approved so far by the United States Food and Drug Administration for acute ischemic stroke. Searching for neuroprotective agents, which can antagonize or reduce injurious biochemical and molecular signal pathways or increase or enhance the protective pathways, are considered a major promising strategy for the treatment of acute ischemic stroke. Therapeutic trials of these neuroprotective agents proved in animal experiments have not yet shown therapeutic benefits in human [2], [6]. Numerous clinical trials failed in past decades because either these agents showed no protective effects in patients or their toxicity/side effects cannot be tolerated by patients. Searching for neuroprotective agents with minimal side effects from natural sources like herbs or plants probably represent an ideal strategy to develop safe and effective agents for stroke treatment.

Carvacrol (CAR), a monoterpenic phenol, is naturally occurring in various plants belonging to the family Lamiaceae. It is abundant in the essential oil fraction of oregano and thyme [7]. CAR has been widely used both as a food or food additive in the food industry for long time. In recent years, its multiple functions were well-studied in different fields. More and more data indicated that CAR has bactericidal activity [8], fungicidal activity [9], [10] and insecticidal activity [11]. Many studies also demonstrated its therapeutic potential on different diseases. Two studies indicated its anti-tumor activity *in vitro* and *in vivo* [12], [13]. In the central nervous system, CAR was regarded as a potential drug for Alzheimer's disease due to its inhibitory effect on acetylcholinesterase (AChE) activity by using phenolic hydroxyl group of CAR to bind to AChE and leading to a loss of function of AChE [14], [15], [16]. In addition, CAR was found to have an antidepressant-like effect in mice by affecting the dopaminergic system [17].

A recent study showed that CAR has the ability to protect liver against ischemia/reperfusion (I/R) injury in rats [18]. These results suggest that CAR, a plant-derived essential oil, may play a role to protect against cerebral I/R injury. Considering CAR's neuroprotective potential and safety to human, in our study, we examined the neuroprotective effect of CAR against cerebral ischemia/reperfusion injury by using the middle cerebral artery occlusion (MCAO) model in mice.

## Materials and Methods

### Animals

Male ICR mice, 23–28 g, were purchased from SLAC Company (Shanghai, China). All animal procedures were approved by the University Committee on Animal Care of Soochow University.

### Experimental groups

Animals were randomly divided into four groups (*n* = 6–10, each): (I) vehicle-treated group (sham); (II) CAR-treated group (CAR group); (III) vehicle-treated I/R group (I/R group); (IV) CAR-treated I/R group (I/R+CAR group). In I/R+CAR group, the animals were divided into several subgroups: pretreatment group (2 h before ischemia), post-treatment 0 h, 2 h, 4 h, 6 h, and 7 h groups. The animals were administered either intracerebroaventricularly (i.c.v.) 10 µg CAR in 10 µl saline (Peptide International Inc, Japan) in the CAR-treated groups or 10 µl vehicle (saline) in the vehicle-treated groups or intraperitoneally (i.p.) 5, 25 and 50 mg/kg body weight CAR or saline.

### The middle cerebral artery occlusion (MCAO) model

MCAO was induced by using an intraluminal monofilament as described before [19]. Briefly, the mouse was anesthetized by chloral hydrate at a dose of 350 mg/kg body weight (i.p.). The right common carotid artery (CCA), the right external carotid artery (ECA) and the internal carotid artery (ICA) were exposed through a ventral midline neck incision. The ECA was ligated with a silk suture at 2 mm distal from the ECA–CCA branch and then cut distal from the ligated point. A silk suture was looped around the CCA and twisted to block blood flow from the CCA. A small incision was performed on the ECA 1.5 mm distal from the ECA–CCA branch. A 6-0 nylon monofilament (Ethilon, Ethicon Inc) coated with silicon resin (Heraeus, Kulzer, Germany) was introduced through the incision into the right CCA and advanced 9–11 mm distal to the carotid bifurcation until a faint resistance was felt for temporary occlusion of the middle cerebral artery. Reperfusion was achieved by withdrawing the suture after MCAO for indicated time (30 min or 75 min) to restore blood supply to the MCA territory. The silk suture looped around the CCA was removed and the neck incision was closed. The sham group underwent the same surgical procedure except that the monofilament was introduced into the external carotid artery but not advanced. Body temperature was maintained at 36.5–37.5°C by means of a heating blanket and a lamp throughout the procedure from the start of the surgery until the animals recovered from anesthesia.

To monitor occlusion and reperfusion, the local cerebral blood flow was measured using a laser-doppler blood flowmeter (Periflux 5010, PERIMED, Sweden) positioned at 1 mm posterior and 3 mm lateral to Bregma.

### Intracerebroventricular administration

For the injection of CAR or saline into the lateral ventricle contralateral to the ischemic side, a small burr hole was made in the parietal region (0.5 mm posterior and 1.0 mm lateral to the Bregma on the left side). A 28G needle on a syringe was inserted into the left lateral ventricle (2.5 mm in depth), and 10 µl of CAR or saline was injected in 10 min.

### Neurological deficit scoring evaluation

Neurological deficits were evaluated according to the following graded scoring system 24 h after the MCAO. The modified scoring system was based on a 5-point scale system described previously [20] : 0, no deficit; 1, flexion of the contralateral torso and forelimb; 2, turning to the ipsilateral side when held by tail; 3, leaning to affected side; 4, no spontaneous locomotor activity. If no deficit was observed after MCAO, the animal was removed from further study.

### Detection of infarction volume

After neurological evaluation, all animals were anesthetized and decapitated, and the brains were removed. The brains with subarachnoid hemorrhage and/or clot formation in the MCA were eliminated from the analysis in this study. All brains were sliced into 1 mm sections. Slices were incubated for 30 min in a 0.2% solution of 2,3,5-triphenyltetrazolium chloride (TTC; Sigma, St. Louis, Missouri) at 37°C and then fixed in 10% buffered formaldehyde solution. For analysis, the sections were photographed by a high-resolution digital camera (Nikon Coolpix L110). The cross-sectional area of infarction in the right MCA territory of each brain slice was determined with a computerized image analysis system (AlphaEase Image Analysis Software V 3.1.2). The total mean infarct area of each section was calculated as the average of the area on its rostral and the caudal surface. The hemispheric lesion volume was calculated as described previously [21].

### Western blot analysis

The animals were sacrificed at indicated time. The ischemic brains were quickly removed and ischemic area brain tissues were collected. Proteins were extracted from brain tissue and protein concentrations were then determined. Proteins (30–60 µg) were loaded on a 12% sodium dodecyl sulfate-polyacrylamide gel electrophoresis (SDS-PAGE) and then electrotransferred to nitrocellulose membrane. Blots were blocked with PBST (3.2 mM Na_2_HPO4, 0.5 mM KH_2_PO4, 1.3 mM KCl, 135 mM NaCl, pH 7.4, 0.1% Tween-20) containing 5% nonfat dry milk for 2 h at 4°C, and then incubated with primary antibodies overnight with shaking at 4°C. After washing, blots were incubated with HRP-conjugated secondary antibody in blocking solution for 1.5 h, and developed by the ECL chemiluminescence system (Thermo Company,West Chester, Pennsylvania, USA) and captured on autoradiographic films (Kodak Company, Rochester, New York, USA). Films were then digitalized with a camera and densitometric analysis of the bands was performed with AlphaEase Image Analysis Software. Primary antibodies were rabbit polyclonal anti-phospho-Akt (Ser473), rabbit polyclonal Akt, rabbit polyclonal cleaved caspase-3 (Cell Signaling Technology Inc., Danvers, Massachusetts), and mouse monoclonal anti-beta-tubulin (Sigma, St. Louis, Missouri).

### Statistical analysis

All data are expressed as mean ± SEM. Differences between groups were determined with Student *t* test for infarct volume; differences among groups were compared by one-way analysis of variance (ANOVA) followed by Tukey's multiple-comparison test if there was a significant difference between groups. *P*<0.05 was considered statistically significant.

## Results

### CAR pre-treatment reduced infarct volume and improved neurological deficits

**Figure 1:**
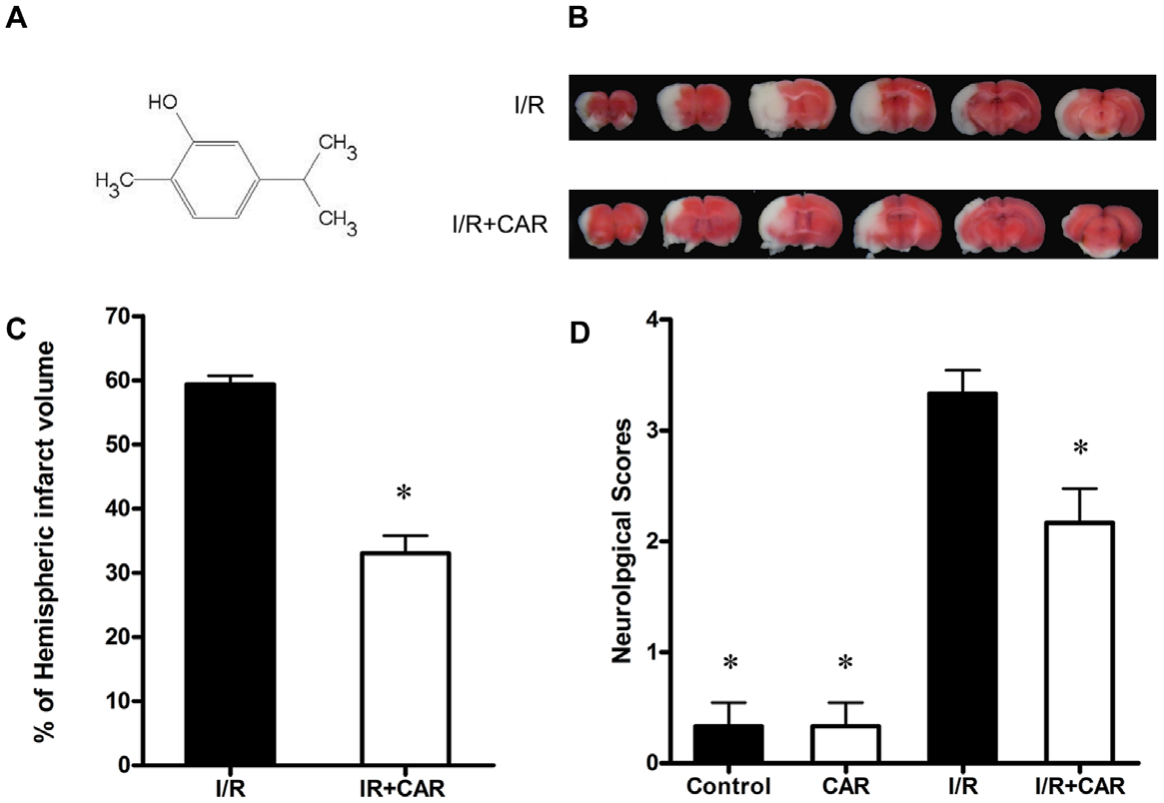
CAR pre-treatment reduced infarct volume and improved neurological deficits after MCAO. Mice were administered (i.p.) with CAR or saline at 2 h before cerebral ischemia. TTC staining and neurological scores were examined after 75 min of ischemia and 24 h of reperfusion. (A)The molecular structure of CAR is shown. (B) The representative TTC-stained coronal sections in vehicle-treated mice and CAR-treated mice (50 mg/kg,i.p.) are shown. (C)Statistical analysis of cerebral infarct volume was performed in I/R group and I/R+CAR group; CAR reduced infarct volume after cerebral I/R injury. (D)Neurological scores were evaluated according to a 5-points scale system. CAR treatment significantly decreased neurological deficits. Bars represent mean ± SEM of 6 brains. *, *P*<0.05 versus vehicle-treated I/R group.

The molecular structure of CAR was shown in Figure 1A (5-isopropyl-2-methylphenol). To examine the protective effect of CAR on cerebral ischemia/reperfusion injury, we performed TTC staining after 24 h of reperfusion and 75 min of MCAO. Effects of CAR and vehicle on infarct volume after the transient MCAO are presented in Figure 1B. There was no detectable infarction in sham group and CAR group, but a large infarct volume was observed in I/R group (Figure 1B). CAR pre-treated mice in I/R+CAR group had a total infarct volume of 33.03±2.75%, which was significantly lower than that in I/R group animals (59.40±1.3%; *P*<0.01) (Figure 1C). CAR markedly reduced infarct volume by approximately 44.4% compared with I/R group.

Neurological scores were examined after 75 min of MCAO and 24 h of reperfusion. Evaluation showed no significant neurological deficits in sham group and CAR group mice, while severe neurological deficits were observed in the I/R group. The mice in I/R group consistently showed circling movements, severe paw flections, or less spontaneous movements. CAR treatment (50 mg/kg body weight i.p.) significantly improved the neurological deficits and decreased neurological score (*P*<0.05, Figure 1D). In I/R+CAR group mice, fewer movement abnormalities in posture and circling movements were found compared with the I/R group mice.

Physiological parameters including heart rate, pH, pO_2_, pCO_2_, temperature, and plasma glucose were measured 30 minutes before and after MCAO. There were no significant differences in various physiologic parameters in the mice treated with CAR or vehicle (Table 1).

**Table 1:** Physiological parameters of control and CAR-treated mice.

	Saline-Treated Mice		CAR-Treated Mice	
	Before Ischemia	After Ischemia	Before Ischemia	After Ischemia
Heart rate, beats/min	538±54	585±47	593±53	567±48
pH	7.28±0.02	7.25±0.06	7.26±0.02	7.23±0.05
pO_2_, mm Hg	92.83±11.13	90.16±8.65	86.75±7.42	88.00±5.36
pCO_2_, mmHg	39.83±4.90	42.36±4.18	45.17±3.89	44.67±3.13
Temperature, °C	36.9±0.14	37.0±0.13	37.0±0.18	37.0±0.16
Glucose, mmol/L	5.70±0.18	5.82±0.25	5.63±0.19	5.78±0.22

Physiological parameters were measured at 30 minutes before ischemia or after 75 min of ischemia and 30 min of reperfusion. Data are expressed as mean ± SEM (n = 6).

### The protection of CAR on infarct volume was in a dose-dependent manner

**Figure 2:**
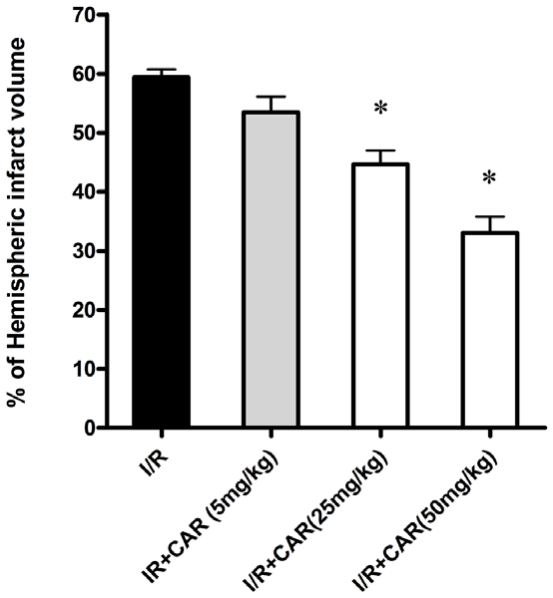
The protection of CAR on infarct volume was in a dose-dependent manner. Mice were administered (i.p.) with CAR at doses of 5, 25, and 50 mg/kg at 2 h before ischemia. Cerebral infarct volume was determined by TTC staining after 75 min of ischemia and 24 h of reperfusion. Bars represent mean ± SEM of 6 brains. *, *P*<0.05 versus vehicle-treated I/R group.

To evaluate the protective efficiency of CAR on cerebral I/R injury in the mouse MCAO model, we treated the mice with different doses of CAR (5, 25, and 50 mg/kg body weight, i.p.). TTC staining after 75 min of ischemia and 24 h of reperfusion indicated that CAR pre-treatment at 5 mg/kg body weight did not reduce infarct volume compared with the I/R group (*P*>0.05, Figure 2); however, CAR pre-treatment at 25 and 50 mg/kg body weight demonstrated significant protection on cerebral ischemia/reperfusion injury (*P*<0.05, Figure 2), suggesting the does-dependent protection of CAR on infarct volume in the mouse MCAO model. In the following experiments, we chose the dose of 50 mg/kg body weight to study the protection of CAR.

### CAR post-treatment had protective effects on cerebral I/R injury

**Figure 3:**
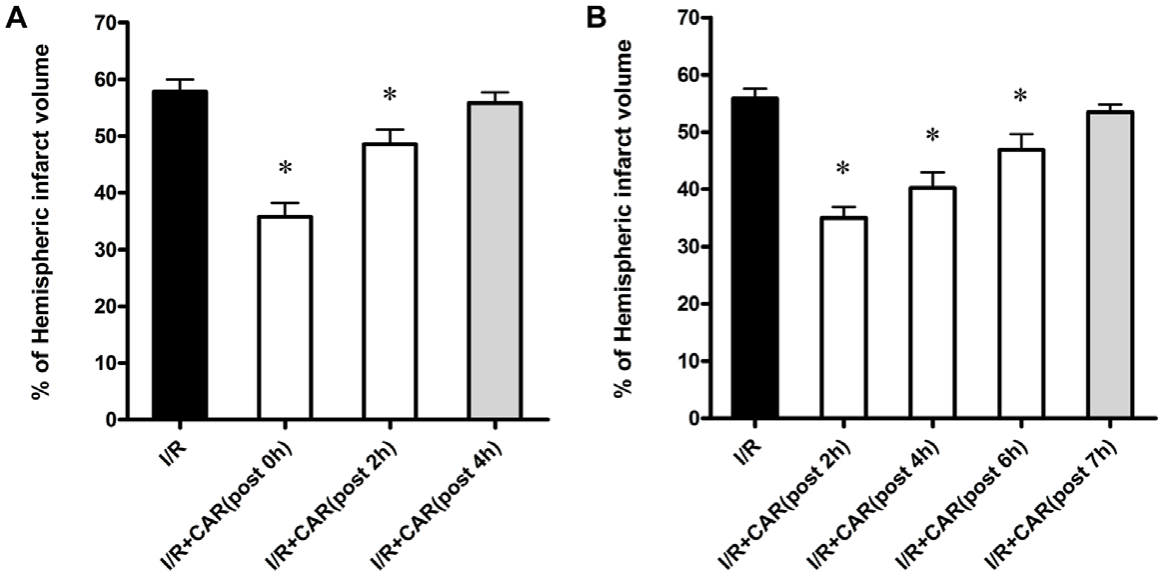
CAR post-treatment reduced infarct volume after cerebral I/R injury. CAR was administered (i.p. and i.c.v.) at different reperfusion times after 75 min of ischemia. TTC staining for cerebral infarct volume was performed after 24 h reperfusion. (A) Mice were administered CAR (50 mg/kg, i.p.) and saline at 0 h, 2 h, and 4 h after reperfusion. CAR had no protection on infarct volume when it was administered at 4 h after reperfusion. (B) Mice were administered with 10 µg CAR (i.c.v.) or saline at 2 h, 4 h, 6 h, and 7 h after reperfusion. CAR still had protective effect when mice treated with CAR after 6 h of reperfusion. Bars represent mean ± SEM of 6 brains. *, *P*<0.05 versus vehicle-treated I/R group.

Because interventions for stroke patients are carried out after a few hours of artery occlusion in the clinical situation, we tested the protective effects of CAR post-treatment on cerebral I/R injury. First, we treated the mice with CAR at the dose of 50 mg/kg body weight (i.p.) at 0 h, 2 h, and 4 h after reperfusion. Our data demonstrated that CAR post-treatment reduced the infarct volume from 57.8±2.2% in the control group to 35.8±2.4% at 0 h after reperfusion and to 48.5±2.6% at 2 h after reperfusion, indicating that CAR post-treatment still had significant protective effects on infarct volume at these two time points (*P*<0.05, Figure 3A); however, at the time point of 4 h after reperfusion, CAR post-treatment did not markedly reduce infarct volume (55.8±1.9%, *P*>0.05, Figure 3A). Due to a short therapeutic window of CAR post-treatment by i.p. administration, we further examined the therapeutic effects of CAR post-treatment by i.c.v. administration at different time points after ischemia. Each mouse was given 10 µg CAR (i.c.v.) at 2 h, 4 h, 6 h, and 7 h after reperfusion. Surprisingly, CAR post-treatment by i.c.v. still protected against cerebal I/R injury even when CAR was administered at 6 h after reperfusion (*P*<0.05, Figure 3B). Our findings indicated that CAR post-treatment has protective effects on infarct volume, but the delivery of CAR should be modified to improve its therapeutic efficiency.

### CAR decreased apoptotic neurons induced by cerebral I/R injury

**Figure 4:**
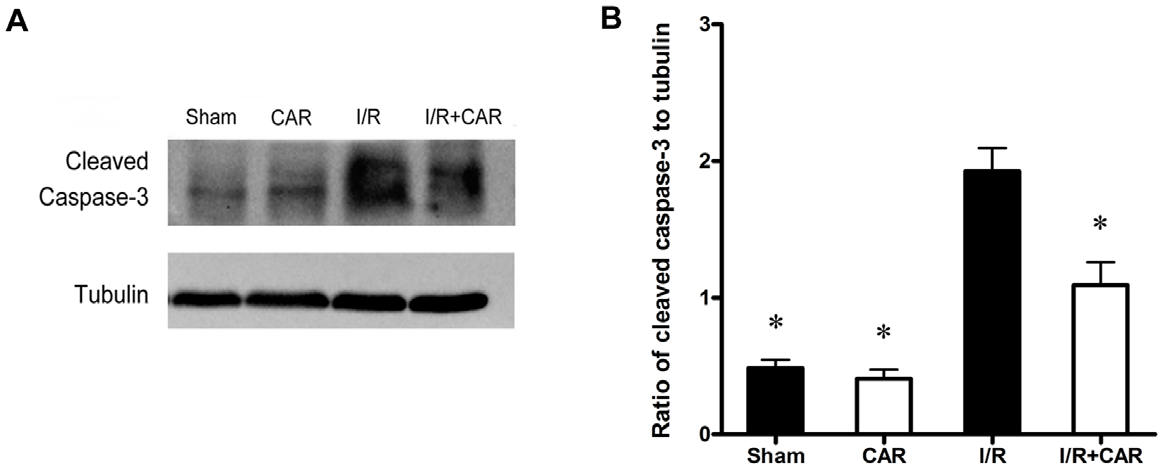
CAR treatment reduced cleaved caspase-3 level after cerebral I/R injury. Mice were treated with CAR (50 mg/kg, i.p.) or saline 2 h before ischemia. Cleaved caspase-3 level was determined by Western blot after 75 min of ischemia and 24 h of reperfusion. (A)The representative photographs show the levels of cleaved caspase-3 and beta-tubulin. Beta-tubulin was used as a loading control. (B)The quantitative analysis indicated that CAR treatment decreased ratio of cleaved caspase-3 to beta-tubulin. Bars represent mean ± SEM for samples from 4 brains in each group. *, *P*<0.05 versus vehicle-treated I/R group.

The increasing evidences showed different kinds of cell death including necrosis, necroptosis, apoptosis, and autophagy in cerebral I/R injury [4]; however, apoptosis and necrosis are still considered as the dominant causes of cell death after cerebral I/R injury. Therefore, we examined whether CAR protects against cerebral I/R injury by blocking the apoptotic neuronal death. Cleaved caspase-3 is regarded as a marker of apoptosis; therefore, we determined the cleaved caspase-3 level after 75 min of ischemia and 24 h of reperfusion by Western blot analysis. We found that I/R injury caused significant increase of cleaved caspase-3 compared with control group and sham group (Figure 4A). CAR treatment decreased the level of cleaved caspase-3 (Figure 4A). Further quantitative analysis showed that CAR treatment markedly decreased cleaved caspase-3 level after MCAO compared with I/R group (*P*<0.05, Figure 4B), suggesting the anti-apoptotic activity of CAR after I/R injury.

### CAR increased phosphorylated Akt (p-Akt) level after cerebral I/R injury

**Figure 5:**
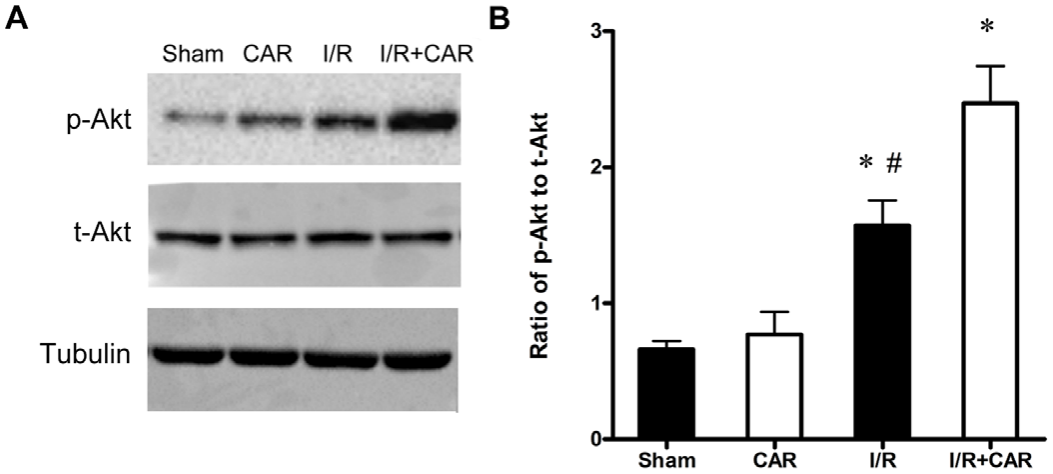
CAR treatment increased the activation of Akt after MCAO. Mice were treated with CAR (50 mg/kg, i.p.) or saline 2 h before ischemia. After 30 min of ischemia and 6 h reperfusion, brain tissues were collected and protein levels were determined by Western blot. (A)The representative photographs show levels of p-Akt, t-Akt, and beta-tubulin. Beta-tubulin was used as a loading control. (B) Quantitative analysis of the ratio of p-Akt to t-Akt was performed. Bars represent mean ± SEM for samples from 4 brains in each group. *, *P*<0.05 versus sham group; #, *P*<0.05 I/R group versus I/R+CAR group.

Since our data demonstrated that CAR prevents neuronal apoptosis after cerebral I/R injury, we explored the signaling pathways that are involved in the neuroprotection of CAR. We examined PI3K/Akt pathway, a survival pathway after cerebral ischemia injury, by detecting p-Akt and total Akt (t-Akt) levels after cerebral I/R injury and CAR treatment. According to our previous study [22], we examined p-Akt level in ischemic brain tissues after 30 min of ischemia and 6 h of reperfusion. Our results indicated that CAR treatment slightly increased p-Akt level in the normal brain without I/R injury but no significant difference was observed compared with sham group (*P*>0.05, Figure 5A). In the mouse brain tissues with I/R injury, the ratio of p-Akt to t-Akt was markedly increased compared with sham group; however, CAR treatment significantly increased p-Akt level in the ischemic brains compared with I/R group (Figure 5A). Quantitative analysis confirmed the difference between I/R group and I/R+CAR group (*P*<0.05, Figure 5B), suggesting that PI3K/Akt pathway is involved in the neuroprotection of CAR on cerebral I/R injury.

### PI3K inhibitor LY-294002 reversed the protection of CAR on cerebral I/R injury

**Figure 6:**
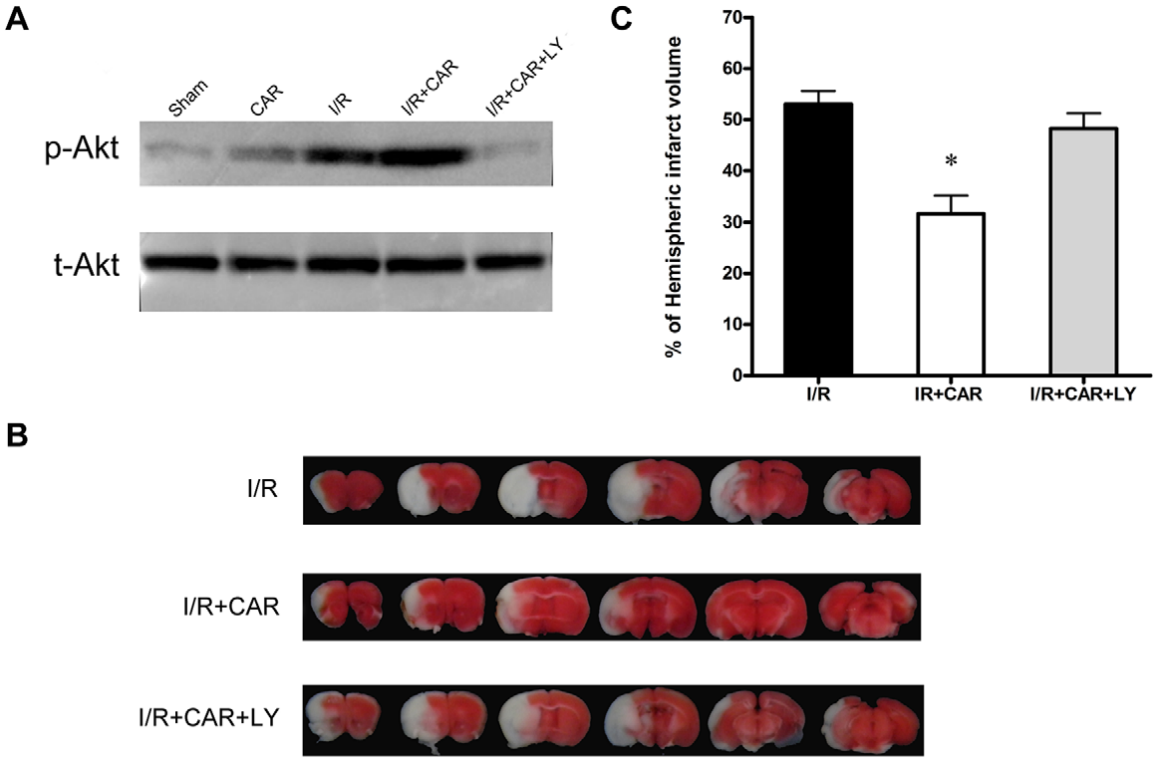
A PI3K inhibitor LY-294002 blocked the neuroprotection of CAR on cerebral I/R injury. Mice were treated with CAR (50 mg/kg, i.p.) or saline 2 h before ischemia and 5 µl LY-294002 (10 mM) was administered (i.c.v.) at 15 min before ischemia. (A) After 30 min of ischemia and 6 h reperfusion, brain tissues were collected and p-Akt and t-Akt levels were determined by Western blot. The photographs show that LY-294002 treatment inhibited the activation of Akt by CAR. (B) After 75 min of ischemia and 24 h of reperfusion, cerebral infarct volume was determined by TTC staining. The representative TTC-stained coronal sections demonstrate that LY-294002 treatment abolished the protection of CAR on infarct volume. (C) Statistical analysis of cerebral infarct volume shows that PI3K inhibitor LY-294002 blocked the protective effects of CAR. I/R+CAR+LY: CAR and LY-294002-treated I/R group. Bars represent mean ± SEM of 6–9 brains. *, *P*<0.05 versus vehicle-treated I/R group.

To further investigate whether the PI3K/Akt pathway mediates the neuroprotection of CAR in cerebral I/R injury, we treated the mice with a PI3K inhibitor LY-294002 (i.c.v., 5 µl 10 mM LY-294002 dissolved in 3% DMSO) at 15 min before ischemia. Western blot analysis showed that the PI3K inhibitor LY-294002 inhibited the increase of p-Akt level induced by CAR treatment and almost restored p-Akt level to basal level (Figure 6A). TTC staining was performed after the treatment with LY-294002 to evaluate its effects on infarct volume. As expected, LY-294002 treatment almost abolished the protection of CAR (Figure 6B). The quantitative analysis of infarct volumes indicated the inhibition of the PI3K/Akt pathway was able to reverse the neuroprotective effect by CAR treatment after MCAO (*P*<0.05, Figure 6C), demonstrating that the PI3K/Akt pathway mediates the neuroprotection of CAR after cerebral I/R injury.

## Discussion

In this study, our data demonstrated for the first time that the food additive CAR has protective effects on cerebral I/R injury in a MCAO model by decreasing infarct volume and reducing neuronal apoptosis (Figures 1, 2 and 4). Post-treatment of CAR still showed significant protection (Figure 3), indicating its potential for the clinical translation from bench research. In addition, our findings showed that PI3K/Akt signal pathway is involved in the protective mechanisms of CAR on cerebral I/R injury (Figures 5 and 6).

CAR is a natural compound extracted from many plants of Lamiaceae family as in the genera Origanum and Thymus. It was widely studied in the past years in different research fields. Studies indicated that CAR has bactericidal [8], fungicidal [9], [10], and insecticidal activity [11] as a food additive. In the medical and biological fields, CAR demonstrated its anti-cancer activity on hepatocellular carcinoma *in vitro* and *in vivo* [12], [13] and its anti-depression activity in rats [17]. CAR also demonstrated its protective effect on inflammation induced by lipopolysaccharide, tumor or autoimmune arthritis [23], [24], [25]. A recent study indicated that dietary CAR supplementation prevents high fat diet-induced obesity by modulating gene expressions that lead to adipogenesis and inflammation [26]. As for I/R injury, Canbek *et al.* demonstrated that CAR protected the biochemical changes in liver caused by ischemia and reperfusion and no hepatotoxicity at the applied dosage of CAR was found [18]. In our study, our findings extended the therapeutic spectrum of CAR and showed that it is a potent protective agent on cerebral I/R injury.

Because the molecular weight of CAR is very small (only 150.2 g/mol) and it has a lipophilic profile, CAR is believed to easily and rapidly cross the blood–brain barrier [27], [28]; but there was no direct evidence to prove that. In this study, we treated the mice intracerebraoventricularly after cerebral I/R injury at different time points. Our data clearly demonstrated the protective effect of CAR even when CAR was administered at 6 h after reperfusion (Figure 3B), suggesting there is an extended therapeutic window for CAR in the MCAO mouse model. While this treatment was administered intraperitoneally, the therapeutic window was significant shorten that CAR had no protective effect when CAR treatment was given at 4 h after reperfusion (Figure 3A). These data suggested that CAR is a high potent neuroprotector on cerebral I/R injury, drug delivery method like intraperitoneal may affect its protective efficiency. The improvement of drug delivery of CAR such as using nanoformulation method will make it easier and higher potent for stroke treatment because the strategy of nanoformulation into liposomes can circumvent solubility, stability, and bioavailability problems [24].

The protective mechanisms of CAR on different diseases were still unclear. Melo *et al.* found that CAR has anti-depressant-like and anxiolytic-like effects in mice by using behavioral test and concluded that the effect of CAR may be mediated by modulating GABAergic transmission and dopaminergic system [17], [29]. Boskabady *et al.* demonstrated the inhibitory effect of CAR on muscarinic receptors in guinea-pig tracheal chains [30]. Similarly, a recent study indicated that CAR is a novel inhibitor of transient receptor potential (TRP) channels in drosophila and mammalian [31]. These studies suggested that CAR may act on membrane receptors to modulate intracellular signal pathways. So far, no data showed whether CAR can directly interact with intracellular proteins or targets by crossing the cellular membrane. However, more and more evidences demonstrated that intracellular signal pathways were changed by CAR treatment [32], [33], [34]. In human hepatoma HepG2 cells, CAR induces cell apoptosis by selectively decreasing phosphorylation of extracellular signal-regulated kinase 1/2 (ERK1/2) and P38 [32]; In human macrophage-like U937 cells, in response to lipopolysaccharide treatment, CAR activates peroxisome proliferator-activated receptors (PPAR alpha and gamma) and suppresses cyclooxygenase-2 (COX-2) mRNA and protein expression [33]. The signal pathways of CAR in vivo were seldom studied. Since our data demonstrated the protective effects, we further examined the effect of CAR on signal pathways after cerebral I/R injury. The phosphatidyl inositide3-kinase (PI3K)/Akt pathway is a critical survival mediator in the signal transduction pathways after cerebral ischemia and the activation of PI3K/Akt pathway is a therapeutic target for stroke [35], [36]. It is not clear whether CAR activates PI3K/Akt pathway after cerebral ischemia injury. In this study, we provided evidences that the PI3K/Akt activation mediates the protection of CAR in the cerebral I/R injury model and the inhibition of PI3K/Akt signal pathway abolishes the protection of CAR (Figures 5 and 6). Although in our study, PI3K inhibitor LY-294002 almost completely blocked the protection of CAR, PI3K/Akt signal pathway may be not the only protective pathway. The antioxidant activity of CAR may also provide a part of neuroprotection because previous studies have confirmed the antioxidant properties of CAR [37], [38]. This inconsistence may be explained by the toxicity of LY-294002 that blocks not only the PI3K/Akt activation by CAR, but also the activation caused by cerebral ischemia injury and even the basal level of Akt.

In summary, CAR provides neuroprotection on infarct volume and neuronal apoptosis in a focal MCAO mouse model. Because CAR is widely used as a food additive and its safety is well studied before [39], [40], it has a high potential to be translated from bench research to clinical trials for stroke therapy.
